# Pesticide Use and Cutaneous Melanoma in Pesticide Applicators in the Agricultural Heath Study

**DOI:** 10.1289/ehp.0901518

**Published:** 2010-02-17

**Authors:** Leslie K. Dennis, Charles F. Lynch, Dale P. Sandler, Michael C.R. Alavanja

**Affiliations:** 1 Department of Epidemiology, College of Public Health, University of Iowa, Iowa City, Iowa, USA; 2 Epidemiology Branch, National Institute of Environmental Health Sciences, National Institutes of Health, Department of Health and Human Services, Research Triangle Park, North Carolina, USA; 3 Division of Cancer Epidemiology and Genetics, National Cancer Institute, Rockville, Maryland, USA

**Keywords:** arsenic, farmers, melanoma, pesticides

## Abstract

**Background:**

Melanoma rates continue to increase; however, few risk factors other than sun sensitivity and ultraviolet radiation (including sun exposure) have been identified. Although studies of farmers have shown an excess risk of melanoma and other skin cancers, it is unclear how much of this is related to sun exposure compared with other agricultural exposures.

**Methods:**

We examined dose–response relationships for 50 agricultural pesticides and cutaneous melanoma incidence in the Agricultural Health Study cohort of licensed pesticide applicators, along with ever use of older pesticides that contain arsenic. Logistic regression was used to examine odds ratios (ORs) and 95% confidence intervals (CIs) associated with pesticide exposure adjusted for age, sex, and other potential confounders.

**Results:**

We found significant associations between cutaneous melanoma and maneb/mancozeb (63 exposure days: OR = 2.4; 95% CI, 1.2–4.9; trend *p* = 0.006), parathion (≥ 56 exposure days: OR = 2.4; 95% CI, 1.3–4.4; trend *p* = 0.003), and carbaryl (≥ 56 exposure days: OR = 1.7; 95% CI, 1.1–2.5; trend *p* = 0.013). Other associations with benomyl and ever use of arsenical pesticides were also suggested.

**Conclusions:**

Most previous melanoma literature has focused on host factors and sun exposure. Our research shows an association between several pesticides and melanoma, providing support for the hypotheses that agricultural chemicals may be another important source of melanoma risk.

The incidence of cutaneous melanoma, the most deadly form of skin cancer, tripled from 1975 to 2006 in the United States (Ries et al. 2006), and it has been estimated that there will be 68,720 new cases of melanoma and 8,650 melanoma deaths in 2009 (Jemal et al. 2009). Light complexion (fair skin, blond or red hair, tendency to burn) and ultraviolet radiation (including sun exposure) are the two major etiologic risk factors for melanoma (Armstrong 1988; Elwood and Jopson 1997). Additional factors include age, family history of melanoma, large numbers of common or atypical nevi, and artificial ultraviolet radiation from tanning lamps (Crutcher and Cohen 1990; Greene 1999; Swerdlow and Weinstock 1998). A variety of other factors have been examined, but no consistent associations have been established.

Several studies of farmers have shown an excess risk of melanoma and other skin cancers (Blair and Zahm 1995; Blair et al. 1992; Spiewak 2001), but it is unclear how much of this is related to sun exposure or to other agricultural exposures. Few studies have assessed pesticides or other chemicals as they relate to melanoma. A recent study showed that arsenic, as measured in toenails, was related to melanoma (Beane Freeman et al. 2004), but no studies have reported on arsenical pesticides. Some evidence also suggests that arsenic may interact with other chemicals, pesticides, and sun exposure (Chen et al. 2006). Overall, the current evidence regarding associations between specific pesticides or chemicals and melanoma is limited.

To examine the potential association between melanoma and pesticides, we examined 50 dose–response relationships for agricultural pesticides and cutaneous melanoma incidence in the Agricultural Health Study (AHS) cohort of licensed pesticide applicators while controlling for known risk factors for melanoma. We also evaluated general classes of pesticides and evaluated other specific pesticides that contain arsenic (reported as ever or never used).

## Materials and Methods

### Cohort enrollment

The AHS is a prospective cohort study of 52,394 private applicators and 4,916 commercial applicators licensed to apply restricted-use pesticides and 32,347 spouses of private applicators from Iowa and North Carolina (Alavanja et al. 1996). For these analyses, we did not include spouses because they were not asked at enrollment about frequency and duration of use of specific chemicals. Recruitment began in December 1993 and continued until December 1997. Private applicators include farmers, farmworkers, and nursery operators, and “commercial” applicators include persons employed by pest control companies or businesses that use pesticides (e.g., warehouse operators, grain elevators). AHS pesticide applicators, who are predominantly white (98%), were enrolled when they completed an enrollment questionnaire at the time of initial licensing or license renewal. Enrolled applicators were also asked to complete a “take-home” questionnaire that sought more extensive information on occupational activities (*n* = 25,291). This study was approved by institutional review boards of the National Cancer Institute, the University of Iowa, and the Battelle Center for Health Evaluation (Durham, NC).

### Questionnaires

The enrollment questionnaire sought information on ever use of 50 pesticides (and detailed exposure information on 22 of these pesticides), crops grown and livestock raised, personal protective equipment used, pesticide application methods used, other agricultural activities and exposures, nonfarm occupational exposures, smoking, alcohol consumption, fruit and vegetable intake, multiple vitamin use, medical conditions, medical conditions in first-degree relatives including a history of cancer, and basic demographic data (all questionnaires are available at http://aghealth.nci.nih.gov/questionnaires.html). The take-home questionnaire included more detailed exposure information for the remaining 28 pesticides on the enrollment questionnaire, along with additional information on personal protective equipment use, dietary and cooking practices, supplemental vitamin use, height and weight (used to calculate body mass index), hours of sun exposure (current and 10 years prior), tendency to burn, hair color, occupational exposures to welding and solvents, and nonfarm jobs. For our purposes, we examined data on pesticides described in detail (22 on the enrollment questionnaire and 28 pesticides on the take-home questionnaire; see Appendix 1). We additionally examined arsenical pesticides checked on the take-home questionnaire in answering the question “What other pesticides have you used frequently (either now or in the past)? (Mark all that you have used).”

### Cohort follow-up

Applicators were linked to cancer registry files in Iowa and North Carolina for case identification from enrollment (i.e., 1993–1997) through 31 December 2005 and to the state death registries and the National Death Index to ascertain vital status (Alavanja et al. 2005). This identified 271 incident cutaneous melanoma cases (hereafter referred to as melanoma) among 56,285 private and commercial applicators after exclusion of subjects with a nonmelanoma cancer diagnosis before enrollment. There were 150 cases of cutaneous melanoma diagnosed after enrollment among applicators without a nonmelanoma cancer diagnosis before enrollment who completed the take-home questionnaire (*n* = 24,704). This included two cases that also had a melanoma diagnosed before enrollment (to increase power). The average length of follow-up among the cohort was 10.3 years.

### Analyses

Dose–response data available for pesticide use included total years of mixing or applying a specific pesticide, days per year of use for an average year, and decade of first use. Lifetime cumulative exposure days were calculated as (application days per year) × (total years of exposure). Lifetime exposure days were then weighted by an intensity score that accounts for pesticide application method and use of personal protective equipment (Dosemeci et al. 2002). Categorical variables were based on the distribution among cases; two exposure categories were created with near equal number of cases and by choosing cutoffs for days of use that correspond to weeks of use (e.g., < 70 days, ≥ 70 days). Only “ever use” data were available for arsenical pesticides (lead arsenate and inorganic and organic arsenic as defined on the questionnaire).

We used AHS data set release AHSREL0803.00 (available on request from AHS). Descriptive frequencies were used to compare cases and noncases regarding sun sensitivity, sun exposure, and obesity (based on body mass index). Unconditional logistic regression was used to examine associations between melanoma and pesticide exposure, adjusted for age categories and sex as well as other variables as indicated. Effect modification by current and past sun exposure (< 6 vs. ≥ 6 hr/day) was examined among pesticides showing a positive association with melanoma. We also examined potential confounding related to known melanoma risk factors, including hours of sun exposure (current and past), tendency to burn, natural red hair color, and body mass index (BMI). For ordered categorical factors, we also present odds ratios (ORs) and 95% confidence intervals (CIs) comparing the highest category with the reference category. To test for linear trend, we fitted a line to the β-coefficients for each category, assuming an equal increase in the ln(OR) for each category level (Breslow and Day 1980).

We limited final analyses to applicators completing the take-home questionnaire to allow for examination of potential confounding effects of melanoma risk factors [sun exposure, tendency to burn, hair color, and BMI, which were only available on the take-home questionnaire]. For the 22 pesticides detailed on the enrollment questionnaire, we compared results using the whole cohort with those for the restricted cohort who completed the take-home questionnaire. Minimal differences were seen.

## Results

Overall, 271 incident melanoma cases were reported among all applicators. There were 150 incident melanomas among 24,704 applicators who completed the take-home questionnaire; this included two applicators diagnosed with melanomas both before and after enrollment. None of the controls had a reported melanoma before enrollment. Table 1 compares melanoma cases and noncases in the cohort by host factors associated with melanoma (Dennis et al. 2008), including sun sensitivity, sun exposure, and body mass index at 20 years of age (which were only available on the take-home questionnaire). Among sun sensitivity factors recorded at baseline, red hair had the strongest association with melanoma. Sun exposure was not linearly related to melanoma in this cohort of pesticide applicators. Compared with other cohort members at enrollment (mean age = 48, median = 47), melanoma cases tended to be older (mean age = 57, median = 59) and have a higher BMI based on weight at age 20 (Table 1). Ethnicity (1% Hispanic), education (19% college graduates), marital status (84% married), and height did not vary by case status.

The 50 pesticides examined for dose–response associations (listed in the Appendix) comprised 18 herbicides, 22 insecticides, 6 fungicides, and 4 fumigants. We found no associations with overall herbicide, insecticide, fungicide, or fumigant use or with the pesticide chemical classes phenoxy herbicides, triazine herbicides, organochlorine insecticides, or organophosphate insecticides (data not shown). Melanoma risk showed a dose–response association with carbamate pesticides overall (trend *p* = 0.032; data not shown because this was the result of the associations with carbaryl and benomyl, two of four carbamate pesticides). Table 2 reports the four specific pesticides that showed a dose–response association with melanoma among applicators. All four pesticides had detailed dose information only on the take-home questionnaire; thus, results in Table 2 are restricted to the take-home questionnaire. None of the 22 pesticides detailed on the enrollment questionnaire was associated with melanoma, compared with 4 of the 28 pesticides detailed on the take-home questionnaire. In an analysis of the 22 pesticides from the enrollment questionnaire, which we restricted to applicators that also completed the take-home, results were similarly negative (data not shown). Although overall fungicide use did not appear to be related to melanoma, two of six fungicides, benomyl and maneb/mancozeb, had significant dose–response associations with melanoma. We also found increased ORs for two insecticides (carbaryl and parathion). Among the cohort members, exposure to carbon tetrachloride was uncommon, with only two cases reporting > 7 days of application over their lifetime. We found no associations with the organochlorine pesticides aldrin, chlordane, dieldrin, dichlorodiphenyltrichloroethane (DDT), heptachlor, lindane, or toxaphene. We found no effect modification of the association with pesticides by sun exposure.

We specifically examined pesticides with arsenic content (Table 3). The crude OR for ever versus never use of lead arsenate was 2.1 (95% CI, 1.1–3.9), but after adjusting for age and sex, ever use of lead arsenate insecticide was not associated with melanoma risk. Ever use of inorganic arsenic herbicides showed a significant association with cutaneous melanoma, but only 44 applicators reported use (OR = 5.4; 95% CI, 1.3–22.9; adjusted for age and sex). None of the few applicators who reported use of organic arsenic herbicides was a melanoma case (data not shown). Exposure to any of these three arsenical pesticides was associated with melanoma similar to lead arsenate (Table 3).

Table 4 reports modification by lead arsenate crop insecticide for the association between melanoma and the pesticides shown in Table 2. Two were significantly modified by use of lead arsenate crop insecticide, whereas parathion was nonsignificantly modified. For all three pesticides (benomyl, maneb/mancozeb, and parathion), we found higher ORs for melanoma (ORs > 6.0) among those who had used arsenical pesticides. These effects could not be explained by age. The carbaryl and melanoma association showed no modification by lead arsenate.

## Discussion

In this study we examined melanoma risk in relation to occupational exposure to pesticides among pesticide applicators in Iowa and North Carolina. The chemical subcohort approach, used in other reports, provides information on all (cancer) outcomes associated with a specific chemical and allows the AHS to provide dose–response information that may inform future risk assessments. The case–control approach used here allows us to consider all factors, not just chemicals, associated with a specific cancer such as melanoma. The AHS pesticide applicators were not shown to be at an increased risk of melanoma relative to populations of these two states (Alavanja et al. 2005), but additional evaluations of melanoma are warranted in light of previous literature. Commonly reported risk factors such as sun sensitivity and sun exposure (Dennis et al. 2008) were associated with melanoma in this cohort.

The strongest pesticide associations were with maneb/mancozeb (a dithiocarbamate fungicide) and parathion (an ethyl or methyl insecticide). In addition, dose–response relationships were seen for two (benomyl and carbaryl) of four different carbamate pesticides. Our carbaryl finding supports a previous report from a prospective analysis of carbaryl applicators in this cohort (Mahajan et al. 2007) with 2 additional years of follow-up (36 additional cases among those completing the take-home questionnaires). Another previous report that focused on organochlorine insecticides within the cohort noted an association between melanoma and toxaphene for lifetime exposure but not for intensity-weighted lifetime days of exposure (Purdue et al. 2006), a measure that takes into account factors such as protective clothing that may modify exposure. We did not see an association with melanoma cases diagnosed through 2005 and toxaphene for intensity-weighted lifetime days of exposure. The data suggested a possible association between melanoma and arsenical pesticides. Although arsenic exposure was limited, arsenical pesticides appeared to modify the effect of benomyl and maneb/mancozeb pesticides independent of age.

The hypothesis that melanoma may be related to pesticides stems from the relationships among epidermal melanocytes, nevi, and the development of melanoma. Dermatitis related to pesticide exposure was described in 1921 (McCord et al. 1921). Other skin diseases or irritations related to pesticides have been reported, including a case report of erythema multiforme related to parathion (Spiewak 2001). A review of 12 studies of farmers found that 8 showed an excess risk of melanoma (7 for other nonmelanoma skin cancers) (Blair and Zahm 1991; Spiewak 2001), but it is unclear how much of this is related to sun exposure compared with pesticides or other exposures. A study of white Ranch Hand Vietnam veterans found an increased risk of melanoma related to dioxin exposure and herbicide exposure (Akhtar et al. 2004). An additional report of an increased standardized incidence ratio for melanoma among Pan Britannica Industry’s pesticide factory workers suggests that pesticides are related to the development of melanoma (Wilkinson et al. 1997). A more recent study found an association with cutaneous melanoma and a longer duration of residential pesticide use (Fortes et al. 2007). They found that the most common compounds for indoor pesticides used in these residents included pyrethroids and carbamates. Additional evidence has shown that pesticides, carbon tetrachloride, and formaldehyde are related to increased risk of intraocular melanoma (Holly et al. 1996). We had too few cases of intraocular melanoma to examine this association.

We did not find other analytic studies that have reported an association with maneb/mancozeb or parathion and melanoma. In this large cohort of pesticide applicators, we only found about 7% of applicators had applied these pesticides; thus, the exposure rate in the general population is likely to be low. However, a study of banana plantation workers in Costa Rica reported an increased standardized incidence ratio for melanoma (Wesseling et al. 1996). Chemicals used on bananas include maneb, mancozeb, and benomyl, along with dibromochloropropane, chlorothalonil, and formaldehyde (Wesseling et al. 1996). They saw that the risk of melanoma also increased with the number of years of employment at banana plantations. This provides further evidence of the potential association between melanoma and maneb/mancozeb and benomyl. For parathion, we did not find any study directly linking it with melanoma. Nevertheless, a laboratory study of sunscreen found that those containing the physical ultraviolet absorbers titanium dioxide or zinc oxide enhance the transdermal absorption of parathion (Brand et al. 2003). In our study, when we further adjusted levels of parathion associated with melanoma for sunscreen use, we found no differences in the ORs. However, applicators were not asked about the details on types of sunscreen used or frequency or duration of use.

A link between arsenic and cancers of the bladder and lung and nonmelanoma skin cancer is well established. An association between arsenic and melanoma has only been reported in one other study to date, with an OR of 2.1 (95% CI, 1.4–3.3) for the highest quartile of toenail arsenic content (Beane Freeman et al. 2004). Our data support the possible association between melanoma and arsenic that is not explained by age, but the data are limited by the rarity of exposure and lack of assessment of frequency or duration of exposure. The mechanistic pathways of arsenical carcinogenesis may include oxidative stress (An et al. 2004; Shi et al. 2004), ultraviolet enhanced mutagenicity (Chen et al. 2006; Rossman 2003), and genotoxicity or altered DNA repair (Huang et al. 1995; Kochhar et al. 1996; Mahata et al. 2003). Arsenic may also work by an epigenetic mechanism that changes the function of the DNA without affecting the normal DNA sequence. Although many arsenical compounds have been discontinued in the United States, arsenical pesticides are still widely available in some countries, and some farms have leftover supplies that continue to represent some potential risk (Reigart and Roberts 1999). Several studies of humans have shown an association between nonmelanoma skin cancer and heavy arsenic exposure via drugs, drinking water with a high arsenic content, or the occupational environment (Chen et al. 1985; Guo et al. 2001; Hsueh et al. 1995, 1997; International Agency for Research on Cancer 1998; Karagas et al. 2001; Pesch et al. 2002; Tseng 1977). Most published studies examining arsenic exposure and skin cancer risk originate from Taiwan, Bangladesh, or China. Among these studies, only one specifically mentioned examining melanoma and did not find an association (Guo et al. 2001); however, melanoma is rare in Chinese populations. An interaction has been demonstrated in one cross-sectional study where the risk of skin lesions associated with various levels of arsenic exposure was greater in those with excessive sun exposure (Chen et al. 2006). We did not see an interaction with sun exposure in our data, but we had limited power to examine this.

The AHS has several strengths, including a prospective design, comprehensive pesticide exposure assessment, completeness of follow-up, and high participation rates. Previous analyses have shown that AHS applicators completing the take-home questionnaire were similar to those who completed only the enrollment questionnaire, with the exception that those completing the take-home questionnaire tended to be older (Tarone et al. 1997). Our analyses (data not shown) of melanoma in association with pesticides detailed on the enrollment questionnaire showed magnitudes for all who completed the enrollment questionnaire similar to those when such analyses were restricted to subjects who also completed the take-home questionnaire. A comparison of the incident cutaneous melanoma cases reported in the overall cohort (those completing the enrollment questionnaire) and those who completed the enrollment and the take-home questionnaire showed similar distributions by histologic site and body site (Dennis et al. 2008). Additionally analyses showed ORs similar to other studies for known sun sensitivity risk factors for melanoma. A limitation of this study was the small number of subjects who applied some of the pesticides, thus limiting the power of some analyses at this time.

Sun exposure, perhaps the strongest risk factor for melanoma, is difficult to capture via questionnaire. Because farmers spend a great deal of time in the sun, we cannot rule out the possibility that these pesticides-specific results are driven by sun exposure. However, results deferred for pesticides within a specific class, and within the limits of small numbers, were similar in Iowa and North Carolina. Furthermore adjusting for owning the farm or farm size (which might affect time outdoors) did not alter these findings. In addition, we had insufficient information on lifelong crop patterns to assess confounding by other factors potentially related to growing orchard fruits where arsenical pesticides were historically used. Finally, multiple comparisons may be an issue because we initially evaluated 50 pesticides. However, we initially focused on associations at the ≥ 0.01 significance level in the crude analyses (data not shown) and considered biologic plausibility. These results should also be interpreted with regard to their consistency with other studies.

## Conclusions

Increased cutaneous melanoma risk was seen among applicators who had used/applied maneb/mancozeb and parathion, and potentially benomyl as well as lead arsenate, compared with never users of these products. The results are consistent with prior findings of an association between melanoma and arsenic. We observed a significant effect modification when benomyl and maneb/mancozeb users were also exposed to lead arsenate. In addition, our previous observation in the AHS of an association between carbaryl and melanoma was upheld when we added 2 additional years of cases. Most of the previous melanoma literature has focused on host factors and sun exposure, but our study suggests more research is needed on chemicals and other environmental factors that may increase the risk of cutaneous melanoma.

## Figures and Tables

**Table 1:** Associations with cutaneous melanoma for sun sensitivity and sun exposure factors in the Agricultural Health Study among 24,704 pesticide applicators completing the take-home questionnaire.

	Cases (*n* = 150)[*n* (%)]	Noncases (*n* = 24,554)[*n* (%)]	Minimally adjusted [OR (95% CI)]^a^	Adjusted^b^ [OR (95% CI)]
Sun sensitivity factors				
Tendency to burn				
No or mild sunburn	102 (69.4)	18,865 (78.0)	Reference	Reference
Blistering or painful sunburn	45 (30.6)	5,313 (22.0)	1.50 (1.05–2.13)	1.23 (0.84–1.78)
Missing	3	376		

Hair color				
Black/brown/blonde	131 (88.5)	23,093 (96.9)	Reference	Reference
Red	17 (11.5)	744 (3.1)	4.00 (2.39–6.66)	3.69 (2.16–6.32)
Missing	2	717		

Eye color				
Brown/green/hazel	77 (52.0)	12,535 (52.0)	Reference	Reference
Blue/gray	71 (48.0)	11,568 (48.0)	0.92 (0.66–1.26)	0.85 (0.61–1.18)
Missing	2	451		

Sun exposure (hours per day spent in the sun during growing season)				
At enrollment (1993–1997)				
≤ 2 hr/day	12 (8.1)	2,522 (10.5)	Reference	Reference
3–5 hr/day	49 (33.1)	6,685 (27.8)	1.56 (0.83–2.94)	1.56 (0.82–2.95)
6–10 hr/day	72 (48.7)	11,157 (46.3)	1.39 (0.75–2.56)	1.38 (0.74–2.56)
> 10 hr/day	15 (10.1)	3,701 (15.4)	1.00 (0.46–2.13)	1.04 (0.48–2.24)
Missing	2	489		

10 years before enrollment				
≤ 2 hr/day	6 (4.3)	1,392 (6.2)	Reference	Reference
3–5 hr/day	24 (17.0)	4,366 (19.4)	1.37 (0.56–3.37)	1.40 (0.57–3.45)
6–10 hr/day	81 (57.4)	11,670 (51.8)	1.56 (0.68–3.58)	1.54 (0.66–3.54)
> 10 hr/day	30 (21.3)	5,091 (22.6)	1.27 (0.53–3.07)	1.31 (0.54–3.16)
Missing	9	2,035		

Obesity				
BMI at 20 years of age				
< 20 kg/m^2^	9 (7.0)	2,997 (13.9)	Reference	Reference
20–24.99 kg/m^2^	72 (56.3)	12,330 (57.0)	2.19 (1.09–4.39)	2.16 (1.07–4.33)
≥ 25 kg/m^2^	47 (36.7)	6,295 (29.1)	3.38 (1.64–6.94)	3.39 (1.65–6.97)
Missing	22	2,932		

Trend *p*-value			*p* = 0.007	*p* = 0.006

a Adjusted for age at enrollment and sex.

b Adjusted for age at enrollment, sex, tendency to burn, and red hair, unless one of these factors is being evaluated, in which case adjustment is limited to the remaining three factors.

**Table 2:** Associations between cutaneous melanoma and pesticides for 150 melanoma cases within 24,704 pesticide applicators completing the take-home questionnaire in the Agricultural Health Study.

Intensity-weighted lifetime days of exposure	Cases [*n* (%)]	Remaining cohort [*n* (%)]	OR (95% CI)^a^
Benomyl (fungicide)^b^			
No exposure	131 (91.0)	21,699 (93.1)	1.0
< 133 exposure-days	7 (4.9)	1,194 (5.1)	1.0 (0.4–2.2)
≥ 133 exposure-days	6 (4.2)	419 (1.8)	2.8 (1.2–6.5)
Missing	6	1,242	
Trend *p*-value			*p* = 0.061

Carbaryl (insecticide)^b^			
No exposure	64 (45.7)	13,570 (60.3)	1.0
< 56 exposure-days	37 (26.4)	5,001 (22.2)	1.3 (0.9–2.1)
≥ 56 exposure-days	39 (27.9)	3,939 (17.5)	1.7 (1.1–2.5)
Missing	10	2,044	
Trend *p*-value			*p* = 0.013

Maneb/mancozeb (fungicide)^c^			
No exposure	127 (88.2)	21,793 (92.9)	1.0
< 63 exposure-days	8 (5.6)	947 (4.0)	1.6 (0.8–3.4)
≥ 63 exposure-days	9 (6.2)	713 (3.0)	2.4 (1.2–4.9)
Missing	6	1,101	
Trend *p*-value			*p* = 0.006

Parathion (ethyl or methyl) (insecticide)			
No exposure	122 (85.3)	21,730 (93.1)	1.0
< 56 exposure-days	10 (7.0)	899 (3.9)	1.6 (0.8–3.1)
≥ 56 exposure-days	11 (7.7)	709 (3.0)	2.4 (1.3–4.4)
Missing	7	1,216	
Trend *p*-value			*p* = 0.003

a Adjusted for age at enrollment, sex, tendency to burn, red hair, sun exposure (≤ 2 hr/day, ≥ 3 hr/day), and BMI at 20 years of age.

b Carbamate pesticide.

c Dithiocarbamate fungicide.

**Table 3:** Arsenical pesticide exposure and 150 cutaneous melanomas among 24,704 pesticide applicators completing the take-home questionnaire in the Agricultural Health Study.

	Exposed [*n* (%)]		OR (95% CI)	
Arsenic^a^	Cases	Noncases	Crude	Adjusted^b^
Lead arsenate crop insecticide				
Never used	140 (93.3)	23,733 (96.7)		
Ever Used	10 (6.7)	821 (3.3)	2.1 (1.1–3.9)	1.2 (0.6–2.3)

Any arsenic pesticide^c^				
Never used	139 (92.7)	23,680 (96.4)		
Ever used	11 (7.3)	874 (3.6)	2.2 (1.2–4.1)	1.3 (0.7–2.4)

a Based on answers to the question “What other pesticides have you used frequently (either now or in the past)?” on the take-home questionnaire.

b Adjusted for age at enrollment and sex.

c Arsenic pesticides included any exposure to lead arsenate crop insecticide, inorganic arsenic herbicide, or organic arsenic herbicide.

**Table 4:** Interactions of lead arsenate and specific pesticides on risk of cutaneous melanoma among pesticide applicators completing the take-home questionnaire in the Agricultural Health Study.

	All subjects		Not exposed to lead arsenate		Exposed to lead arsenate		
Pesticide/exposure	Cases/noncases^a^	OR (95% CI)^b^	Cases/noncases^a^	OR (95% CI)^b^	Cases/noncases^a^	OR (95% CI)^b^	*p*-Value for interaction^c^
Benomyl^d^ (fungicide)							
No exposure	131/21,699	1.0 (reference)	128/21,110	1.0 (reference)	3/589	1.0 (reference)	
Any exposure	13/1,613	1.2 (0.7–2.1)	7/1,440	0.7 (0.3–1.6)	6/173	6.7 (1.6–27.0)	*p* = 0.006

Carbaryl^d^ (insecticide)							
No exposure	64/13,570	1.0 (reference)	63/13,444	1.0 (reference)	1/126	1.0 (reference)	
Any exposure	76/8,940	1.5 (1.0–2.0)	67/8,309	1.4 (1.0–2.0)	9/631	1.8 (0.2–14.4)	*p* = 0.835

Maneb/mancozeb^e^ (fungicide)							
No exposure	127/21,793	1.0 (reference)	125/21,235	1.0 (reference)	2/558	1.0 (reference)	
Any exposure	17/1,660	1.5 (0.09–2.5)	9/1,457	0.9 (0.5–1.8)	8/203	10.8 (2.3–51.3)	*p* = 0.005

Parathion (insecticide)							
No exposure	122/21,730	1.0 (reference)	120/21,238	1.0 (reference)	2/492	1.0 (reference)	
Any exposure	21/1,608	1.9 (1.2–3.0)	13/1,331	1.5 (0.8–2.7)	8/277	7.3 (1.5–34.6)	*p* = 0.065

a Total varies based on the number of subjects with missing values for each pesticide.

b Adjusted for age at enrollment and sex using the intensity-weighted lifetime exposure days.

c *p*-Value for a multiplicative interaction term.

d Carbamate pesticide.

e Dithiocarbamate fungicide.

**Appendix 1:** Pesticide frequency and duration data evaluated for associations with melanoma within the Agricultural Health Study, 1993–1997.

Category/questionnaire	Pesticides
Herbicides	
Enrollment	Alachlor, atrazine, cyanazine, dicamba, 2,4-D, EPTC, glyphosate, imazethapyr, metolachlor, trifluralin
Take-home	Butylate, chlorimuron-ethyl, metribuzin, paraquat, pendimethalin, petroleum oil as herbicide, 2,4,5-T, 2,4,5-TP

Insecticides	
Enrollment	Carbofuran, chlorpyrifos, coumaphos, dichlorvos, fonofos, permethrin, terbufos, trichlorofon
Take-home	Aldicarb, aldrin, carbaryl, chlordane, diazinon, dieldrin, DDT, heptachlor, lindane, malathion, parathion, phorate, toxaphene

Fungicides	
Enrollment	Captan, chlorothanil, ziram
Take-home	Benomyl, maneb/mancozeb, metalaxyl

Fumigants	
Enrollment	Methyl bromide
Take-home	Aluminum phosphide, ethylene dibromide, carbon tetrachloride/carbon disulfide

Abbreviations: 2,4-D, 2,4-dichlorophenoxyacetic acid; 2,4,5-T, 2,4,5-trichlorophenoxyacetic acid; 2,4,5-TP, 2,4,5-trichlorophenoxypropionic acid; EPTC, *S*-ethyl dipropylthiocarbamate.
